# No reason to expect large and consistent effects of nudge interventions

**DOI:** 10.1073/pnas.2200732119

**Published:** 2022-07-19

**Authors:** Barnabas Szaszi, Anthony Higney, Aaron Charlton, Andrew Gelman, Ignazio Ziano, Balazs Aczel, Daniel G. Goldstein, David S. Yeager, Elizabeth Tipton

**Affiliations:** ^a^Institute of Psychology, ELTE Eötvös Loránd University, Budapest, 1053 Hungary;; ^b^Department of Economics, University of Stirling, Stirling, FK9 4LA Scotland;; ^c^Marketing Department, Illinois State University, Normal, Illinois 61761;; ^d^Department of Statistics, Department of Political Science, Columbia University, New York, New York 10027;; ^e^Marketing Department, Grenoble Ecole de Management, Grenoble, 38000 France;; ^f^Microsoft Research’s New York City Lab, Microsoft Research, New York, New York 10012;; ^g^Department of Psychology, The University of Texas at Austin, Austin, Texas 78712;; ^h^Department of Statistics and Data Science, Northwestern University, Evanston, Illinois 60208

As policy makers are increasingly interested in implementing nudge-type interventions, it is essential that we understand under what conditions they can improve policy-relevant outcomes to make the best possible use of public resources. For that reason, the recently published metaanalysis by Mertens et al. (1) of the choice architecture literature is laudable.

Our reading of the data and analyses, however, is quite different from Mertens et al.’s (1): Nudge interventions may work, under certain conditions, but their effectiveness can vary to a great degree, and the conditions under which they work are barely identified in the literature (2). For example, the authors assume that the nudge literature is impacted by publication bias; that is, larger positive, and statistically significant, comparisons are more likely to be reported. After adjusting for a hypothesized severe to moderate degree of publication bias, their adjusted estimated average effect of nudges is between *d* = 0.08 (severe) and *d* = 0.31 (moderate). Our additional analysis on the same database applying three different bias-correcting methods, compared to the nonadjusted estimate, also led to much smaller effect sizes (Andrews–Kasy, *d* = −0.01, SE = 0.02; weighted average of the adequately powered [WAAP], *d* = 0.07, SE = 0.03; Trim and fill, *d* = 0.08, SE = 0.03) (see also ref. 3). Furthermore, the authors estimate that, even after adjusting for publication bias, the effects of nudge interventions vary considerably across studies. For example, assuming a severe degree of publication bias, 95% of these studies’ effects would be ±1.00 around the average *d* = 0.08 effect, showing large variability, with much of this variability possibly arising from variability in publication bias itself.

Nevertheless, Mertens et al. (1) focus their message on the average effect size estimated without adjusting for publication bias, concluding that “our results show that choice architecture interventions overall promote behavior change with a small to medium effect size of Cohen’s *d* = 0.43” (p. 1). We argue that this effect size is implausibly large, which could be misleading and further strengthen researchers’ and practitioners’ overoptimistic expectations (3, 4) about the impact of nudges. Furthermore, the authors focus their conclusions on this average value and on subgroups, leaving aside the large degree of unexplained heterogeneity (5) in apparent effects across published studies. For example, despite the analyses above being consistent with a large proportion of studies having near-zero underlying effects, the authors conclude that nudges work “across a wide range of behavioral domains, population segments, and geographical locations” (p. 7).

Thankfully, it is because Mertens et al. (1) conducted these analyses and shared their data that we were able to notice these contradictions between findings and conclusions. We argue that, as a scientific field, instead of focusing on average effects, we need to understand when and where some nudges have huge positive effects and why others are not able to repeat those successes (2, 4, 5). Until then, with a few exceptions [e.g., defaults (6)], we see no reason to expect large and consistent effects when designing nudge experiments or running interventions.

## Data Availability

Analysis code is available in Open Science Framework at https://osf.io/bntm6/ (7).
